# Gender equity in neurosurgery requires structural reform

**DOI:** 10.3389/frhs.2026.1850868

**Published:** 2026-07-17

**Authors:** Linda Liverani, Nina Dwumfour-Poku, Silvia D. Vaca, Corinna C. Zygourakis

**Affiliations:** 1 Department of Neurosurgery, Stanford Health Care, Palo Alto, CA, United States; 2 School of Medicine, UT Southwestern, Dallas, TX, United States

**Keywords:** brain drain, gender equity, low- and middle-income countries, neurosurgery, NSOAP, structural reform, women in surgery, workforce

## Abstract

Women remain severely underrepresented in neurosurgery, constituting approximately 7% of the global neurosurgical workforce. Global disparities in women neurosurgeons are shaped by interconnected systems of infrastructure, governance, and education that differ substantially between high income countries (HICs) and low- and middle-income countries (LMICs). While HICs have formalized residency pathways, institutional mentorship frameworks, and professional societies dedicated to gender equity, LMICs face systemic barriers including inadequate surgical infrastructure, limited training capacity, scarce mentorship, and insufficient gender equity policies. Moreover, the brain drain phenomenon disproportionately affects female physicians from LMICs, who migrate to HICs seeking better compensation, safer working conditions, and professional advancement, thereby reducing local neurosurgical capacity. However, comparisons of a country's economic status and global gender gap index—a measure of gender disparity—to its female neurosurgical workforce have revealed that disparities remain in HICs and there are lessons to be learned from progress in LMICs. Africa demonstrates the highest proportional representation of women neurosurgeons globally at approximately 15% yet has wide inter-country variability and the smallest neurosurgical workforce in the world, making percentages heavily influenced by the presence of even a few women neurosurgeons. Underrepresentation becomes self-reinforcing: women lack advocates and mentors to advance through training pathways and into leadership positions without women in surgical leadership. National Surgical, Obstetric, and Anesthesia Plans (NSOAP) are country-led building blocks aimed at strengthening surgical systems and offer a structured framework to address these inequities systematically.

## Introduction

Neurosurgery remains one of the least gender diverse medical specialties worldwide. Women make up approximately 7% of the global neurosurgical workforce, a proportion that has improved only marginally despite decades of advocacy (1). The barriers to entry and retention are not primarily individual failures but structural ones, rooted in interconnected systems of infrastructure, governance, and education that differ substantially between HICs and LMICs (1, 2).

Given the complex intersectional nature of these factors, gender equity in neurosurgery demands systemic change rather than isolated programmatic solutions. In HICs, formalized residency pathways and professional societies have driven incremental progress, yet structural gaps in compensation, parental leave, and institutional culture persist (3, 4). In LMICs, where over 23,000 additional neurosurgeons are needed to meet current demand, deficits in surgical infrastructure, training capacity, mentorship, and gender responsive governance compound these barriers further (5–7).

The WHO Health Systems Framework and the National Surgical, Obstetrics, and Anesthesia Plan (NSOAP) building blocks offer a practical lens for analyzing these disparities and proposing actionable reforms (6, 8). This perspective examines how structural forces shape the global female neurosurgical workforce, with particular attention to differences between HIC and LMIC settings, the brain drain dynamic, and the self-reinforcing cycle of underrepresentation. It then proposes a reform framework organized around the six NSOAP building blocks.

## Global landscape of gender disparities in neurosurgery

Women neurosurgeons are underrepresented in every world region, though the degree and nature of that underrepresentation vary substantially. In North America, women constitute approximately 14%–17% of neurosurgery residents but only 8%–10% of practicing neurosurgeons (1, 9). European figures are comparable, with some nations reporting higher proportions among trainees than among senior faculty, suggesting pipeline improvement without equivalent leadership advancement at this time (10). Globally, women account for a minority of academic neurosurgeons and are even more underrepresented on editorial boards, society leadership, and among named lecturers (2, 11).

A country's Global Gender Gap Index (GGGI)—a measure of gender disparity in the country—has a modest, but statistically significant association with the number of women neurosurgeons in its workforce (1). This, coupled with the fact that several LMIC have favorable GGGIs suggest that while HIC overall have more neurosurgeons per capita, gender disparities remain and there are lessons to be learned from gender equity efforts in LMICs (1).

Africa presents a paradox. It holds the highest proportional representation of women neurosurgeons globally at approximately 15% yet has wide inter-country variability and operates within the smallest neurosurgical workforce in the world, serving populations with among the highest burdens of neurosurgical disease (1, 12). This paradox does not necessarily reflect advancement but rather the extreme smallness of the total workforce in many African nations, where percentages are heavily influenced by the presence of even a few women neurosurgeons. Absolute numbers remain critically low (13).

In South and Southeast Asia, women neurosurgeons face barriers tied to cultural norms around professional autonomy, on-call schedules incompatible with unpaid domestic labor, and limited institutional support for pregnancy and parenthood (14). Latin America shows emerging evidence that women enter training programs but face disproportionate barriers for academic promotion and leadership (11).

Across all regions, women enter neurosurgery at lower rates than men, face higher attrition throughout training, and are systematically underrepresented in leadership, research output, and academic appointment (1, 2). In LMICs, these dynamics are amplified by resource constraints that affect all trainees but disproportionately disadvantage women (6, 12).

## Structural barriers in HIC and LMIC settings

In HICs, structural barriers to gender equity in neurosurgery are increasingly well-documented. Gender pay gaps persist across surgical specialties, with documented disparities in industry compensation for academic neurosurgeons (3, 15). Parental leave policies at medical institutions frequently disadvantage women: among top ranked US medical schools, only 14.9% offer 12 weeks of fully paid leave for birth mothers, and 43.7% offer no paid leave at all for non-birth parents (4). In Japan, male physicians take parental leave at a rate of 0.05% compared to 4.5% for female physicians, highlighting how the burden of childcare falls disproportionately on women across different national contexts (16). Mentorship programs for women in neurosurgery have historically been inconsistently implemented and rarely formalized within training curricula (17), though ongoing efforts through national organizations such as Women in Neurosurgery (WINS) in the United States have markedly improved the quantity and quality of mentorship opportunities available (18, 19).

Implicit bias shapes selection and evaluation at every career stage. Studies have shown that women trainees face environmental invalidations, defined as systematic aggressions that result in a perception that women do not belong in surgery, and that women surgeons are held to higher standards than their male colleagues to achieve academic promotion (6). The culture of neurosurgery in many HIC institutions continues to implicitly code professional identity around traits perceived as masculine, creating environments that many women experience as unwelcoming and that drive attrition both before and after training completion (6, 20).

In LMICs, these barriers are compounded by fundamental health system failures. Surgical infrastructure is often inadequate for safe training, limiting trainee capacity regardless of gender. Mentorship networks are sparse and informal. Institutional policies protecting pregnant trainees or ensuring equitable promotion are largely absent. Not all challenges in LMICs are gender related. Limited infrastructure, scarce training capacity, and weak workforce planning affect all neurosurgeons, men and women alike. On top of these system-level problems, women face additional barriers seen across regions: pregnancy and motherhood, unequal domestic responsibilities, scarce parental leave, lack of female mentors, and institutional bias. This distinction matters: not every workforce gap in LMICs is a gender issue, but women still carry an extra burden in low resource settings. A 2025 Italian survey by Cappelletto et al. (21) confirms that these gender specific barriers persist even in high income European settings. Participants reported gaps in mentorship, discrimination during training and at work, work family imbalance, and limited visibility in scientific societies. They also called on neurosurgical societies to reward merit rather than tokenistic representation, which directly supports the structural reform proposed here (21).

The combined effect is a progressive filter that disproportionately removes women at each stage of the neurosurgical career pathway, from medical school interest through residency application, training, and academic advancement. In LMICs, this filter operates within a workforce already critically insufficient relative to disease burden (5, 22).

## The brain drain paradox

Brain drain of healthcare workers from LMICs to HICs is a well-documented threat to global surgical equity. The brain drain phenomenon disproportionately affects female physicians from LMICs, who migrate to HICs seeking better compensation, safer working conditions, and professional advancement (2, 23). In Pakistan, 64% of neurosurgery trainees reported intentions to emigrate, driven by limited fellowship opportunities and inadequate research exposure (24). Female physicians face additional push factors including personal safety concerns, lack of career advancement, and unsupportive institutional cultures, alongside pull factors such as research opportunities, mentorship access, and greater professional security in HIC settings (22, 24).

Female neurosurgeons who emigrate from LMICs to HICs represent a dual loss. Their departure removes technical expertise from underserved systems and eliminates precisely the role models most needed to recruit and retain the next generation of women in neurosurgery locally. Gender mediates access to and participation in health worker training, employment, and ultimately migration, yet policy responses rarely consider these gender dimensions (2).

This dynamic is most acute in sub–Saharan Africa and South Asia, where the total neurosurgical workforce is smallest, and female representation is most fragile (1, 12). Retention strategies that fail to address the structural conditions driving emigration cannot succeed. Meaningful retention requires livable working conditions, equitable compensation, functional mentorship, and institutional cultures that support rather than penalize parenthood and professional development (2, 22).

In some instances, international mobility may provide a paradoxical benefit to source countries through return migration of skilled providers who are able to contribute positively to their home country's workforce. Emigration of healthcare workers may also create an incentive for building a skilled workforce, though evidence of return migration is mixed (25–27). An additional unintended consequence to consider is whether diversity, equity and inclusion initiatives in HICs targeted at LMICs, while positively increasing recruitment opportunities for underrepresented groups, may also worsen local shortages of skilled providers in low-resource settings.

## The reinforcing cycle of underrepresentation

The lack of women in surgical workforces creates self-reinforcing cycles. Without visible female role models and leaders, women in medical training are less likely to consider neurosurgery as a career. Without women in training programs, there are fewer internal advocates capable of reshaping institutional culture. Without culture change, barriers to recruitment and retention remain intact (20, 23).

This cycle operates across all world regions, but is most entrenched in LMICs, where it intersects with absolute workforce scarcity. When the total neurosurgical workforce is small, the normative influence of existing practitioners is amplified, and the absence of women becomes a more powerful deterrent to female entry.

Breaking this cycle requires deliberate disruption at multiple points simultaneously. Single point interventions, such as mentorship programs without accompanying policy reform or compensation equity without institutional culture change, produce limited and often temporary effects (8, 24). Structural reform must be systemic and coordinated across the health system building blocks that govern training, financing, and workforce planning.

## A framework for structural reform

National Surgical, Obstetric, and Anesthesia Plans (NSOAPs) are health policy frameworks developed to strengthen surgical, obstetric, and anesthesia care across multiple domains of the health system in LMICs, including workforce, service delivery, infrastructure, information management, finance and governance (28). These NSOAP building blocks are developed by first performing a comprehensive situational analysis followed by an agreement on priorities by key stakeholders (28). These priorities are then written into a draft that is circulated whereby time-bound indicators, governance structures and chains of accountability are defined by stakeholders (28). Cost of implementation is estimated and evaluated based on attainability before plan is escalated through a formal ministry of health process (28). They are then integrated into a nation's existing health care system and provide practical architecture for translating strategic reform into concrete action (7, 28, 29). Currently, 48 countries have implemented NSOAP including Zambia, Nigeria, Ethiopia, Ecuador, and Brazil (30).

Strategic interventions require simultaneous action across multiple domains: infrastructure investment in surgical facilities, implementation of anti-discrimination and transparent compensation policies, formalization of mentorship programs, integration of gender responsive workforce planning into national health policies, and cultural transformation that acknowledges unpaid household labor inequities (7, 18, 28, 29). Gender equity cannot advance in neurosurgery without explicit institutional commitment, and NSOAPs are the most direct mechanism through which national governments can operationalize that commitment (28, 29).

Key NSOAP elements include training a sustainable workforce, setting physician density targets, strengthening service delivery and health information systems, ensuring access to neurosurgical equipment, and protecting communities from catastrophic surgical costs (Table 1). Each of these apply directly to reducing gender inequities in global neurosurgical systems which have been introduced in numerous studies (7, 28, 29).

**Table 1:** NSOAP building blocks linked to gender equity initiatives in neurosurgery.

NSOAP building blocks	Gender equity initiatives
Leadership and Governance	Integrate recruitment and retention strategies for female neurosurgeons into national surgical policy mediated by each nation's ministry of health.
Finance	Allocate portion of national healthcare spending to ensuring adequate paid parental leave, funded through payroll taxes. Recommend also codifying paid parental leave into law to offer job protection for parents, similar to most HICs.
Information Management	Collect data on surgical outcomes, workforce composition, surgical volume as well as information on local workforce shortages, and rural compared to urban access to neurosurgical care. Particularly, information revealing workforce gender gaps can help drive downstream policy.
Service Delivery	Collect data on physician-patient gender concordance and care quality/outcomes to inform policy.
Workforce	Promote equal surgical opportunities and pay across genders. Expand training and leadership pathways for women neurosurgeons.
Infrastructure and Products	Ensure equal access to surgical supplies, equipment and facilities.

Metrics for implementation success of NSOAP based on expert consensus include geospatial access (proportion of a country's population within 2 h access of a facility capable of providing surgical and anesthesia care for cesarean section, laparotomy, and treatment of open fracture), workforce (number of each of surgery, obstetric, or anesthesia providers actively practicing, per 100,000 population), surgical volume (number of procedures done in an operating room, per 100,000 population per year), perioperative mortality (deaths from all causes, before discharge in all patients who have received anesthesia for a procedure done in an operating room, divided by the total number of procedures, per year, expressed as a percentage), and catastrophic expenditure (percentage of population at risk of catastrophic expenditure if they were to require surgery) (31). Additionally, in a neurosurgical and gender equity context these success metrics can be expanded to include common neurosurgical procedures such as decompressive craniotomy as well as markers of gender equity including the annual rate of recruitment and retention of female trainees, promotion to leadership/representation in professional societies, access to parental leave, and availability of mentorship.

Finally, the single largest barrier to NSOAP implementation and retention is funding, with many NSOAPs remaining underfunded despite implementation (32, 33). Avenues to address systemic underfunding include a focus on local capacity building as opposed to international missions and increasing political drive for investment in surgery by framing it as a cost-effective and necessary health intervention (32, 34).

## Discussion

Gender inequity in neurosurgery is structural in origin and requires structural solutions. The evidence converges on a single conclusion: isolated voluntary measures are insufficient. Disparities affecting women globally must be addressed through reform aligned with the building blocks that govern health systems (8, 20).

Several limitations apply to this analysis. Workforce data on women in neurosurgery remain incomplete, particularly in LMICs where registry systems are weak (29). The evidence base for specific interventions, including mentorship program design and parental leave policy effects on training outcomes, draws primarily from HIC settings and may not transfer directly to LMIC contexts without adaptation (17, 35). The gendered dimensions of health worker migration from LMICs remain poorly studied, limiting the ability to design gender-responsive retention strategies (5). Future research should prioritize intervention evaluation in LMIC settings and develop validated measures of gender equity for inclusion in national surgical planning frameworks.

The NSOAP framework offers a mechanism for structured, measurable reform. The global neurosurgical community must place gender equity as a key component of capacity building. Structural reform is inseparable from the mission of expanding neurosurgical access. A workforce that systematically excludes women cannot adequately serve the populations with the greatest burden of neurosurgical disease (5, 29).

## Conclusion

Gender disparities in neurosurgery are not random. They follow clear patterns and are maintained by structural failures in health systems. Geography, cultural norms, weak mentorship, and brain drain reinforce each other across regions. The result is the same everywhere: women are kept out of a field they are fully able to lead. The NSOAP building blocks offer a clear framework to change this. National governments, neurosurgical training programs, and neurosurgical societies should implement these strategies as a means to incorporate gender equity in workforce planning, training, and governance.

## References

[1] Vaca SD Theologitis M Zygourakis CC. Women neurosurgeons worldwide: characterizing the global female neurosurgical workforce. Neurosurgery. (2024) 94(5):916–25. 10.1227/neu.0000000000002796 38084996

[2] Bourgeault IL Runnels V Atanackovic J Spitzer D Walton-Roberts M. Hiding in plain sight: the absence of consideration of the gendered dimensions in source country perspectives on health worker migration. Hum Resour Health. (2021) 19(1):40. 10.1186/s12960-021-00571-6 33761939 PMC7992834

[3] Lyons NB Bernardi K Olavarria OA Shah P Dhanani N Loor M. Gender disparity among American medicine and surgery physicians: a systematic review. Am J Med Sci. (2021) 361(2):151–68. 10.1016/j.amjms.2020.10.017 33526213

[4] Slostad J Jain S McKinnon M Chokkara S Laiteerapong N. Evaluation of faculty parental leave policies at medical schools ranked by US news & world report in 2020. JAMA Netw Open. (2023) 6(1):e2250954. 10.1001/jamanetworkopen.2022.50954 36689228 PMC9871796

[5] Dewan MC Rattani A Fieggen G Arraez MA Servadei F Boop FA. Global neurosurgery: the current capacity and deficit in the provision of essential neurosurgical care. J Neurosurg. (2019) 130(4):1055–64. 10.3171/2017.11.JNS171500 29701548

[6] Sprow HN Hansen NF Loeb HE Wight CL Patterson RH Vervoort D. Gender-based microaggressions in surgery: a scoping review of the global literature. World J Surg. (2021) 45(5):1409–22. 10.1007/s00268-021-05974-z 33575827

[7] Sonderman KA Citron I Mukhopadhyay S Albutt K Taylor K Jumbam D. Framework for developing a national surgical, obstetric and anaesthesia plan. BJS Open. (2019) 3(5):722–32. 10.1002/bjs5.50190 31592517 PMC6773655

[8] Stephens EH Heisler CA Temkin SM Miller P. The current status of women in surgery: how to affect the future. JAMA Surg. (2020) 155(9):876–85. 10.1001/jamasurg.2020.0312 32639556

[9] Woodrow SI Gilmer-Hill H Rutka JT. The neurosurgical workforce in North America: a critical review of gender issues. Neurosurgery. (2006) 59(4):749–58. 10.1227/01.NEU.0000232671.44297.DF 17038940

[10] Corr F Zeitlberger AM Hostettler IC Firtinidou A Schulz E Neidert MC. Gender disparity and workforce trends in German neurosurgery: a longitudinal national analysis. Brain Spine. (2025) 5:105596. 10.1016/j.bas.2025.105596 40994522 PMC12454873

[11] Campos LN Naus A Rangel AG Brandão GR Faria I Pierre TAJ. Women representation on editorial boards in Latin America journals: promoting gender equity in academic surgery, anesthesia, and obstetrics. World J Surg. (2023) 47(4):845–53. 10.1007/s00268-022-06872-8 36587176

[12] Karekezi C Thango N Aliu-Ibrahim SA Bechri H You Broalet EM Bougrine M. History of African women in neurosurgery. Neurosurg Focus. (2021) 50(3):E15. 10.3171/2020.12.FOCUS20905 33789234

[13] Dewan MC Baticulon RE Rattani A Johnston JM Warf BC Harkness W. Pediatric neurosurgical workforce, access to care, equipment and training needs worldwide. Neurosurg Focus. (2018) 45(4):E13. 10.3171/2018.7.FOCUS18272 30269579

[14] Pahwa B Kalyani M Jain I Bhattacharjee S. Will you choose neurosurgery as your career? An Indian female medical student perspective. J Clin Neurosci. (2022) 105:1–8. 10.1016/j.jocn.2022.08.015 36049362

[15] Ngaage LM Harris C Gao C Puthumana J Crabill GA Baglien B. Investigating the gender pay gap in industry contributions to academic neurosurgeons. World Neurosurg. (2019) 130:516–22.e1. 10.1016/j.wneu.2019.06.145 31254703

[16] Fukami K. Gender gap in parental leave among physicians in Japan. Womens Health Rep (New Rochelle). (2024) 5(1):385–92. 10.1089/whr.2023.0126 39035150 PMC11257144

[17] Mahendran GN Walker ER Bennett M Chen AY. Qualitative study of mentorship for women and minorities in surgery. J Am Coll Surg. (2022) 234(3):253–61. 10.1097/XCS.0000000000000059 35213486

[18] Plonsker JH Benzil D Air EL Woodrow S Stippler M Ben-Haim S. Gender equality in neurosurgery and strategic goals toward a more balanced workforce. Neurosurgery. (2022) 90(5):642–7. 10.1227/neu.0000000000001910 35311744

[19] INS White Paper Committee, Benzil DL Abosch A Germano I Gilmer H Maraire JN. The future of neurosurgery: a white paper on the recruitment and retention of women in neurosurgery. J Neurosurg. (2008) 109(3):378–86. 10.3171/JNS/2008/109/9/0378 18759565

[20] Shi HH Westrup AM O’Neal CM Hendrix MC Dunn IF Gernsback JE. Women in neurosurgery around the world: a systematic review and discussion of barriers, training, professional development, and solutions. World Neurosurg. (2021) 154:206–13.e18. 10.1016/j.wneu.2021.07.037 34280544

[21] Cappelletto B Rispoli R Peretta P Valentini LG Garozzo D Fornari M. Women in neurosurgery aim for recognition of merit, not tokenism: insights from an Italian survey. Front Surg. (2025) 12:1594731. 10.3389/fsurg.2025.1594731 40529890 PMC12171119

[22] Alokozay E Haider E Waseem N Alokozay N. Addressing global disparities in neurosurgical workforce and access to care. Chin Neurosurg J. (2025) 11(1):30. 10.1186/s41016-025-00419-1 41261410 PMC12632040

[23] Hagander LE Hughes CD Nash K Ganjawalla K Linden A Martins Y. Surgeon migration between developing countries and the United States: train, retain, and gain from brain drain. World J Surg. (2013) 37(1):14–23. 10.1007/s00268-012-1795-6 23052799

[24] Shakir M Altaf A Irshad HA Khan MAA Enam SA. Brain drain: a cross-sectional study evaluating migration intentions of neurosurgery trainees in Pakistan. Asian J Neurosurg. (2024) 19(2):160–7. 10.1055/s-0043-1778086 38974436 PMC11226287

[25] Nwadiuko J Switzer GE Stern J Day C Paina L. South African physician emigration and return migration, 1991–2017: a trend analysis. Health Policy Plan. (2021) 36(5):630–8. 10.1093/heapol/czaa193 33778873

[26] Eaton J Baingana F Abdulaziz M Obindo T Skuse D Jenkins R. The negative impact of global health worker migration, and how it can be addressed. Public Health. (2023) 225:254–7. 10.1016/j.puhe.2023.09.014 37949017

[27] Poppe A Wojczewski S Taylor K Kutalek R Peersman W. The views of migrant health workers living in Austria and Belgium on return migration to sub-Saharan Africa. Hum Resour Health. (2016) 14(Suppl 1):27. 10.1186/s12960-016-0129-4 27381038 PMC4943491

[28] Citron I Jumbam D Dahm J Mukhopadhyay S Nyberger K Iverson K. Towards equitable surgical systems: development and outcomes of a national surgical, obstetric and anaesthesia plan in Tanzania. BMJ Glob Health. (2019) 4(2):e001282. 10.1136/bmjgh-2018-001282 31139445 PMC6509614

[29] Truché P Shoman H Reddy CL Jumbam DT Ashby J Mazhiqi A. Globalization of national surgical, obstetric and anesthesia plans: the critical link between health policy and action in global surgery. Global Health. (2020) 16(1):1. 10.1186/s12992-019-0531-5 31898532 PMC6941290

[30] Anyomih TTK Agbeko AE Aregawi AB Chu K Crawford R Harrison EM. Systematic review of the lancet commission on global surgery indicators with quality assessment of modelled estimates. Br J Surg. (2026) 113(3):znaf289. 10.1093/bjs/znaf289 41779633 PMC13016785

[31] Davies JI Gelb AW Gore-Booth J Martin J Mellin-Olsen J Åkerman C. Global surgery, obstetric, and anaesthesia indicator definitions and reporting: an Utstein consensus report. PLoS Med. (2021) 18(8):e1003749. 10.1371/journal.pmed.1003749 34415914 PMC8415575

[32] Nepogodiev D Picciochi M Ademuyiwa A Adisa A Agbeko AE Aguilera M-L. Surgical health policy 2025–35: strengthening essential services for tomorrow's needs. Lancet. (2025) 406(10505):860–80. 10.1016/S0140-6736(25)00985-7 40675172

[33] Seyi-Olajide JO Anderson JE Williams OM Faboya O Amedu JO Anyanwu SN. National surgical, obstetric, anaesthesia and nursing plan, Nigeria. Bull World Health Organ. (2021) 99(12):883–91. 10.2471/BLT.20.280297 34866684 PMC8640693

[34] Alayande BT Bekele A. Five years to the finish line: progress and unintended consequences since the lancet commission on global surgery. Br J Surg. (2025) 112(15 Suppl):xv3–9. 10.1093/bjs/znaf205 41369657

[35] Myrcha P Siripurapu V Gloviczki M Dua A Gloviczki P. Women surgeons: barriers and solutions. Ann Vasc Surg. (2024) 105:325–33. 10.1016/j.avsg.2024.02.024 38599486

